# Personal Guides: Heterogeneous Robots Sharing Personal Tours in Multi-Floor Environments

**DOI:** 10.3390/s20092480

**Published:** 2020-04-27

**Authors:** Igor Rodriguez, Unai Zabala, Pedro A. Marín-Reyes, Ekaitz Jauregi, Javier Lorenzo-Navarro, Elena Lazkano, Modesto Castrillón-Santana

**Affiliations:** 1 Faculty of Informatics, University of Basque Country (UPV/EHU), 20018 Donostia, Spain; igor.rodriguez@ehu.eus (I.R.); unai.zabalac@ehu.eus (U.Z.); ekaitz.jauregi@ehu.eus (E.J.); 2 University Institute of Intelligent Systems and Numeric Applications in Engineering, Campus de Tafira, Las Palmas de Gran Canaria, University of Las Palmas de Gran Canaria, 35017 Las Palmas, Spain; pedro.marin102@alu.ulpgc.es (P.A.M.-R.); javier.lorenzo@ulpgc.es (J.L.-N.); modesto.castrillon@ulpgc.es (M.C.-S.)

**Keywords:** social service robots, distributed robotic system, face re-identification, neural networks

## Abstract

GidaBot is an application designed to setup and run a heterogeneous team of robots to act as tour guides in multi-floor buildings. Although the tours can go through several floors, the robots can only service a single floor, and thus, a guiding task may require collaboration among several robots. The designed system makes use of a robust inter-robot communication strategy to share goals and paths during the guiding tasks. Such tours work as personal services carried out by one or more robots. In this paper, a face re-identification/verification module based on state-of-the-art techniques is developed, evaluated offline, and integrated into GidaBot’s real daily activities, to avoid new visitors interfering with those attended. It is a complex problem because, as users are casual visitors, no long-term information is stored, and consequently, faces are unknown in the training step. Initially, re-identification and verification are evaluated offline considering different face detectors and computing distances in a face embedding representation. To fulfil the goal online, several face detectors are fused in parallel to avoid face alignment bias produced by face detectors under certain circumstances, and the decision is made based on a minimum distance criterion. This fused approach outperforms any individual method and highly improves the real system’s reliability, as the tests carried out using real robots at the Faculty of Informatics in San Sebastian show.

## 1. Introduction

According to the International Federation of Robotics, service robots aim to assist humans in performing useful tasks and can be categorized according to the type of interaction they are able to demonstrate [1]. There are many applications for service robots [2]: in health centers [3]; in warehouses, moving material from one location to another in distribution centers [4]; in retail stores, guiding the customers to the products of their choice [5]; offering coffee to clients [6]. It is becoming increasingly frequent to read about robots acting as shopping assistants [7] and as domestic helpers [8]. They can potentially be used for nursing-care situations [9] and assisting the elderly at home [10].

One of the first service robot applications was that of tour-guiding in museums. Since Minerva [11], several robots have been deployed for such tasks. Tour-giving robots heavily rely on autonomous navigation, but also require many other capabilities and even social aptitudes. Guide robots can be considered as social service robots because they need to exhibit some social capabilities while interacting with the human clients in a natural way. There is no doubt that people verification plays an important role during social interactions. We mostly recognize people from their faces, though other characteristics contribute to the recognition process [12].

In [13], a heterogeneous robot navigation system was presented, called GidaBot, that relied on robot communication to fulfill guiding tasks cooperatively on different floors. Besides being fully distributed, the peculiarity of the system was that robots were restricted to navigate only on one floor, and multi-robot communication and cooperation were used to give tours of multiple locations on different floors. A critical system shortage is the lack of visitor identification capability. As guiding tasks are shared among robots, users need to be re-identified when they are picked up by a new robot to be able to give personalized services. In this paper, we present a step forward, integrating a face recognition module into GidaBot, which allows each robot to identify the user that demanded the tour. In this way, personalized tours are not disturbed by new users. It is worth noting that since visitors are casual users, no long-term recognition is required and that only face matching is needed, with the added complexity that the images compared are captured by different robots endowed with the same camera placed at a different height on each robot and the varying environmental conditions (dependent on the current location of each robot). Thus, a face recognition module is needed that is capable of coping with the variability that the particular guiding system imposes.

The contributions of this paper are as follows:

1. First, a standard face recognition approach is evaluated in re-identification and verification experiments with real data taken from the robotic platforms for which the face recognition module is being developed. Face embeddings are used as face feature vectors, and face similarity is calculated by measuring the Euclidean distance between those vectors. Different offline experiments validate the obtained model, in keeping with recent literature.
2. In the real robotic system, an interaction protocol is introduced to ensure the capture of the subject’s face, thus reducing face pose challenges.
3. In addition, an opportunistic strategy is adopted including face detection expert fusion. A set of face detectors providing a slightly different normalized face and therefore resulting face embeddings. The novelty relies in the adoption of a minimal distance approach in the face embedding representation space, which reduces the dependency of each single face detector strength and weakness.
4. Both the interaction protocol and the fusion approach are validated in the real tour guide system using three robots with different morphologies and moving on different floors of a building. The re-identification task is thus validated in a system where each time the pair of images to be matched is taken by different robots located on different floors and in different locations.

The rest of the paper is structured as follows: Section 2 describes the relevant literature about tour guide robots, on the one hand, and about face recognition in social service robots, on the other. Next, Section 3 briefly summarizes the main components of the GidaBot system. Section 4 follows with the development and performance of the face re-identification system. Section 5 describes how the multi-robot system is modified to cope with the user identification task, and Section 6 details the experiments performed to test the viability and evaluate the performance of the whole system. Finally, in the last section, conclusions are described and challenges outlined.

## 2. Related Work

### 2.1. Tour Guide Robots

As mentioned in the Introduction, the robot Minerva [11] was the pioneer of tour-guiding robots. It acted in the Smithsonian’s National Museum of American History in Washington for several weeks, and it is by far the most cited one. Minerva was part of the TOURBOT and WebFAIR projects [14] in which the guiding tasks were extended with the possibility of teleoperating the robot via the Internet and providing web users with the possibility of seeing exhibitions. Since then, many museums have offered guided robotic tours. For instance, Robovie was used at the Osaka Science Museum to assist visitors [15]. KTBot [16] (a robot designed by Tekniker Research Technology Centre (https://www.tekniker.es/en) provided guiding services at the Eureka Science Museum of San Sebastian. Robotinho showed similar capabilities at the Deutsches Museum of Bonn [17]. All those examples showed similar capabilities, and the development focused on a single robot working in a unique floor scenario.

A rather different approach can be found in [18], where the navigation capabilities of CoBot were improved by cooperating with the visitor and helping each other to fulfill the task. A different type of collaboration among robots can be found in [19]. These robots share profiles and tour information so that when successive robots meet the group, they can optimize the relevance of the information they give. Tours are limited to a single floor. Museums are not the only scope of tour-guiding robots. Glas et al. [20] proposed a framework based on a network robot system for different tasks such as guiding customers in a shopping mall. Environmental sensors, central planning servers, and cloud-based resources were used, and robots acted as the visible elements of the network.

A crucial challenge to be met is the usefulness of those robots (initially intended to be used in single floor buildings) in public places with multiple floors. There are few alternatives that cope with this extra complexity. For example, the PR2 robot robot Charlie [21] was able to localize and operate the lift to perform a delivery task. The elevator was located on the map, and the robot used its manipulators inside it. A similar work extended to multiple robots was proposed for the GuideBot tour guide [22] and BellBot hotel assistant [23] systems. The two systems made use of the elevators to navigate between floors. They needed a central server and adopted a master-slave architecture, where each robot was not aware of any other agent.

Robots that get into lifts are supposed to have the necessary abilities to locate, detect, and interact with the lift interface, inside and outside, to execute precise actions. However, this kind of technology is not yet available in common buildings. The lack of proper actuators can be overcome by interacting with humans as CoBot does [18,24]. This symbiotic collaboration approach has been further expanded to a homogeneous team of up to four robots that are also able to perform delivery tasks [25].

Parra et al. [13] proposed an alternative approach to the previous robot-human communication. Robots communicate with robots in GidaBot, without any centralized server that controls the multi-robot system. In GidaBot, a heterogeneous robot team cooperates, sharing services, in a completely decentralized mode. Although several platforms are needed, robot navigation is more secure (robot paths do not collide), and several tours can be run concurrently. In this way, the lift remains available for people involved or not in the guided tour and for people with reduced mobility [13].

### 2.2. Face Recognition in Social Service Robots

Undoubtedly, service robots increase sociability when they can recognize people. People recognition has been addressed by the mobile robotics research community for a long time. Despite the fact that face recognition produced notable results, little attention has been paid to the real applications of face re-identification in social robots, as mentioned in [26]. The research presented in [27] was probably one of the first attempts. In it, Wong et al. divided the human recognition problem into several steps and used a non-supervised feature extraction algorithm, together with a two-layer back-propagation neural network trained to distinguish faces and attach an identity to the face image. The system was tested on a Cybermotion K2A robot with an accuracy of 70%.

Different features can be used to identify people. In [28], three soft biometrics (clothing, complexion, and height) were compared to be used by a humanoid robot in a social setting for person identification. However, in our scenario, soft biometrics is not available because the robot only sees the user’s face and the top of the shoulders. Although other sensorial inputs like voice and touch might be used, vision-based face recognition is one of the most reliable methods to recognize humans [29].

Vision-based methods for face identification can be holistic (use the face region as the input), feature-based (use the local features such as eyes), or hybrid (those that combine the whole face with local features). Regardless of the approach, sensitivity to pose and illumination variations entails the major challenge, especially when the face identification is performed in mobile robots [30].

Face recognition in service robots is especially crucial in robotic surveillance systems. In [31], the authors analyzed the performance of a PCA Eigenfaces-based face recognition system in a mobile robot to determine the optimal speed of the robot in a dynamic environment. Vinay et al. [32] proposed a binary feature detector and descriptor named SPHORB (Spherical Oriented Fast and Rotated Brief) as an effective face recognition technique for robotic surveillance systems, but no robot was used during the experiments presented. The approach proposed in [33] looked for long-term face re-identification. The authors used Bayesian inference-based face classification, and the system associated predefined people data with online learned faces using face features. The approach was applied to a network of fixed RGB-D cameras responsible for identifying a person. Then, the result of the identification was sent to a small robot that approached the person to perform a simple task. They did not have to cope with moving capturing systems, nor with environmental variability.

Due to the recent advances in object detection and recognition (e.g., [34,35]), traditional methods (e.g., Eigenfaces or descriptors such as SIFT/SURF/HOG) have been now superseded by deep learning approaches. Jiang et al. [36] proposed a deep convolutional neural network system, combining a region proposal network and FaceNet [37] for home robot applications. The goal differed from our approach in that they attempted to identify eight human faces, whereas in the system we present, the goal is to match new faces once and again. In [38], Almeida measured the robustness of a facial recognition system in the robot MASHI. The robot was assumed to assist users in several social tasks; however, the users were known in advance, and the face recognition system was designed as a supervised classification problem. The robot was tested at different distances from the users, but it did not move at all. Mohammad and Rad [12] tried to identify people using neural networks through different characteristics of the face, body, and voice of the person. They combined multi-modal signals with the purpose of person recognition in a social robot, but only off-line experiments were performed; no real application was shown. Wang et al. [26] proposed a multiple face re-identification system. Similar to us, they used face embeddings, but combined with unsupervised clustering, achieving a remarkable performance. However, the final goal of integrating the system in a single robot, TERESA, remains as further work, and only performance values over two databases were reported.

Further, regarding research prototypes, some commercially available social robots have face recognition capabilities. For instance, Softbank’s robots are well known by the robotics community, and their NAOqi API offers face detection and tracking. Recently, Softbank announced a collaboration with everAI (a USA company delivering mission-critical face recognition technology that excels at accuracy) (https://www.paravision.ai/#about-us). Anki’s Cozmo tiny robot is another example of a social robot with facial recognition integrated. The faces to be recognized must be shown to the robot so that it learns to classify them.

Our approach differs in some aspects from previous systems. The face recognition module is evaluated in real-world conditions where robots operate in a realistic interaction scenario with a highly dynamic environment. The lighting and capture conditions vary considerably depending on the robot location. Besides this complex scenario, images taken from different heights and perspectives and at different spots should be compared. Moreover, no memory is kept; visitors are casual users, and only one-shot recognition is required; no knowledge of the faces to be recognized is available for acquiring the face recognition model. As far as we know, no face recognition approach as the one proposed here has been included in social service robots navigating in dynamic environments.

## 3. GidaBot Tour Guide System Description

This section attempts to give a general overview of GidaBot, the multi-robot distributed guide system designed to share navigation tasks in multi-floor environments. For further details, readers should refer to [13]. In short, GidaBot allows two operation modes: single target mode and tour mode (which relies on the latter). Independent of the mode, during the guiding processes, the robots must cope with different situations. If the user is already on the floor that corresponds to the desired destination, only one robot will guide the user from the beginning to the end of the navigation task. This scenario is the one that typically occurs in common guide robots. On the contrary, if the user and the desired goal are on different floors, two robots will be involved, the one located on the floor where the tour starts and the robot placed on the floor where the navigation ends. In this situation, robot communication is crucial, and the first robot will send the user to the destination floor (from the stairs or elevator) where the second robot will pick the user up and bring him/her to the final goal. From now on, we will use the term “floor change” to refer to this situation.

**Figure 1:**
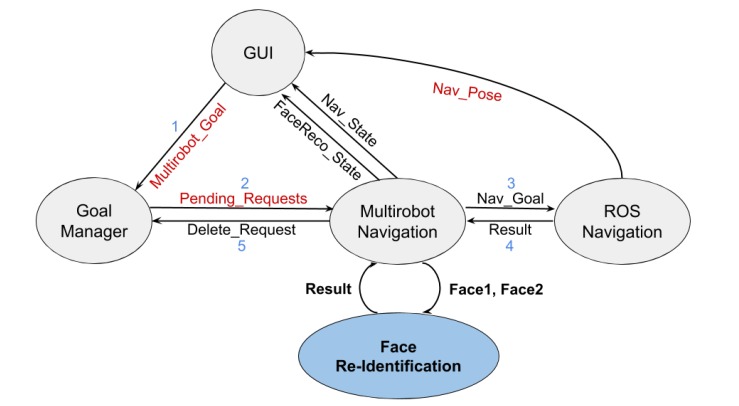
Communication among system nodes.

There are three main components of the system, implemented as a set of ROS packages (see Figure 1):

1. The graphical user interface.
2. The multi-robot navigation subsystem that relies on the ROS navigation stack, composed by two nodes: multirobot_navigation node and the goal_manager node.
3. The communication subsystem.

Note that all elements depicted in Figure 1 are executed in every robot. The numbers on the links reflect the communication flow among the nodes during the navigation task, while the text highlighted in red refers to the messages shared among robots. The node filled in blue corresponds to the face recognition subsystem that will be described later on in Section 5.

### 3.1. The Graphical User Interface

**Figure 2:**
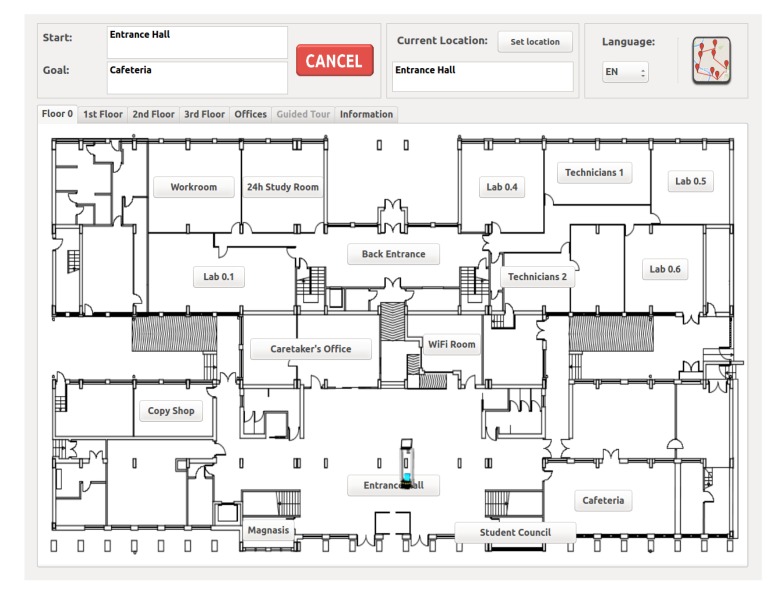
Main view of the GUI. The visible tab corresponds to the ground floor where a PeopleBot robot is present at the main entrance waiting for the visitors (reproduced with permission from Springer Nature under License Number 4792610973165).

Figure 2 shows a general overview of the interface developed. This interface allows users to interact easily and intuitively with the robots. Note the different tabs that comprise the main window. Each tab displays the floor’s blueprint containing some buttons with the available destinations together with information related to the current navigation task. Some extra information is also displayed in the Information tab like robot velocity and battery level. Again, the reader is referred to [13] for more precise information.

GidaBot’s graphical interface has been designed to offer the user:

- The option to choose among the different operation modes available (single target mode and tour mode).
- The option to select the language of the system (Basque, Spanish, and English).
- Visual detailed information about the different locations and easy definition and management of tours.
- Information about the current state of the robots during any guiding task.
- The option to cancel the task at any time.

Additionally, each time the user wants to set a goal, the GUI informs her/him of the pending requests of the robots guiding her/him. If she/he thinks it will take long to wait, the visitor can always choose to cancel the task.

A video available on RSAIT’s Youtube channel (https://www.youtube.com/watch?v=i1UtxrGieks) shows the whole tour recorded during a guided tour composed of eight goals and covering the four floors of the faculty.

### 3.2. Multi-Robot Navigation

GidaBot was developed using ROS and, thus, makes use of the ROS navigation stack. Basically, all robots use the particle filter-based Augmented Monte Carlo Localization (AMCL) algorithm for localization and Dijkstra’s planner to search for available paths to the goals. The global and local navigation parameters were adapted to each robot’s needs. For the time being, the map of each floor does not include the internal description of the rooms, and the guide system limits the robot navigation to the doors that give access to the target locations.

It is worth summarizing here the description of the two ROS nodes implemented that make the multi-robot navigation possible:

- The goal_manager node: this node is in charge of processing the list of pending request, which includes navigation goals. In case a robot receives more than one request, these are queued in order of arrival and managed using a First-In-First-Out (FIFO) queue. Thus, pending goals are processed in the same order they are requested.
- The multirobot_navigation node: this main node receives the list of the pending navigation goals, processes this information, and then sends the robot to the pertinent place. Once a robot has finished processing a request, if its queue is not empty, it will start navigating to the next goal (the first in the queue). It also provides the GUI with all the information required to keep the user informed: the trip, the robot’s navigation state, and the actions that need to be performed, as well as the failures detected by the navigation subsystem that are exchanged.

### 3.3. Communication Subsystem: Messages and Broadcasting

The approach employed for the robots’ communication relies on a specific information exchange. ROS facilitates message definitions and communication among nodes via topics or services. The problem arises when the messages need to be broadcast to different machines, i.e., robots. ROS is not designed for distributed systems in which information must be shared among all the entities. Fortunately, the ROS community offers a few alternatives that facilitate the distribution of topics among machines in ROS, such as the multimaster package, which enables communication between two ROS masters. What it does is register topics or offer services at a different ROS master and/or subscribe to topics or call to services of the same master.

**Figure 3:**
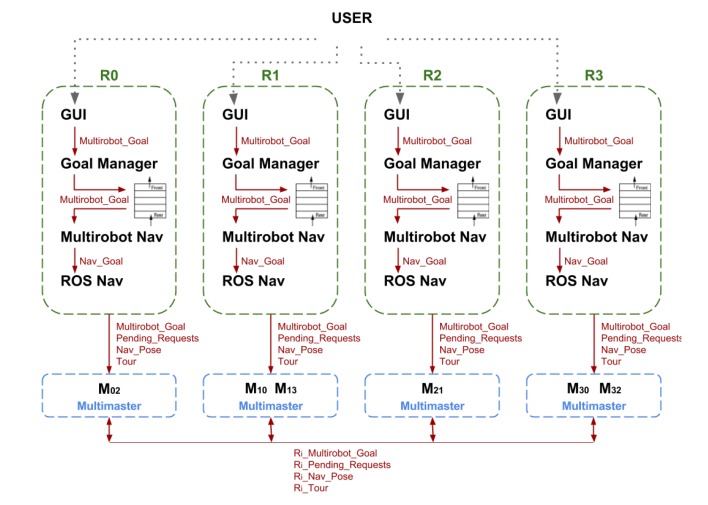
Multi-robot system architecture. $M_{ij}$ denotes that the multi-master facilitates communication between masters *i* and *j*, i.e., robots *i* and *j* (reproduced with permission from Springer Nature under License Number 4792610973165).

In GidaBot, each robot runs its own ROS master, and all robots are interconnected on a complete graph network, resulting in a low latency messaging platform. The multimaster node must be executed just in one of the two masters we want to connect. This means we need $\frac{n(n-1)}{2}$ multi-masters, where *n* is the number of robots. Before running the node, the foreign master must be specified, together with the local publications we wish to share and the foreign publications to be received. Figure 3 shows the system architecture for four robots. In this case, the system requires a definition of six multi-masters in order to have full intercommunication among all the robots.

## 4. Development of the Face Re-Identification/Verification Module

The face recognition process is comprised of several steps. Once the image is captured, the face detection and facial landmarks’ estimation are applied. As the eye locations serve to perform face alignment, only those faces whose facial landmarks are detected are further processed. In the experiments described below, we made use of two different face detectors, applying first the fastest, and the second one only if no detection was reported. This strategy is certainly expensive in terms of computational cost. In scenarios with hardware restrictions, a single and light face detector should be adopted. A possible approach in such scenarios is the combination of a classical Viola and Jones face detector [39], combined with Viola and Jones-based eye detectors [40] to reduce false positive detection.

The fastest one is the DLIB [41] implementation of the Kazemi and Sullivan fast face detector based on the Histogram of Oriented Gradients (HOG) and linear SVM [42]. The resulting detection is used as input to a face pose estimator based on 68 facial landmarks pre-trained with the iBUG 300-W dataset [43]. The alternative facial detector is the Multi-Task Cascaded Convolutional Network (MTCNN) [44]. For any detector, the returned eye locations are used to perform normalization including a light face alignment and posterior cropping. This process involves a rotation and scaling, obtaining a final cropped image of 160×160 pixels where the eyes are located respectively in positions (52, 52) and (104, 52).

Once the detected face was normalized, we used FaceNet embeddings as the feature vectors [37]. FaceNet maps facial images to a Euclidean space, where distances serve as a measure of face similarity. This approach has been extensively used recently [36,45,46,47,48,49,50] and is currently among the state-of-the-art in face recognition benchmarks, with the advantage that it provides a compact feature vector of just 128 values where Euclidean distance may be applied.

The training process of FaceNet is based on the triplet loss that compares a baseline input (anchor) with respect to a positive input (identity same as the anchor) and the negative input (identity different from the anchor). Thus, the training set will be made up of triplets ($x_{i}^{a},x_{i}^{p},x_{i}^{n}$), $x_{i}^{a}$ being the anchor sample and $x_{i}^{p}$ and $x_{i}^{n}$ the positive and negative samples, respectively. Each sample is the input of each of the three branches that configure FaceNet and that share the same weights. The triplet aims to minimize the distance of the anchor and positive inputs and maximize the distance between the anchor and negative inputs. Formally, triplet loss is defined as:

(1) $${Loss}_{T}=\sum_{i=1}^{N}\left[|\right|f_{i}^{a}-f_{i}^{p}{\left|\right|}_{2}^{2}-\left|\right|f_{i}^{a}-f_{i}^{n}{\left|\right|}_{2}^{2}{+\alpha ]}_{+}$$

where α is a margin distance parameter and $f_{i}^{a}$, $f_{i}^{p}$, $f_{i}^{n}$ are the embedding vectors extracted from the three branches of the network, the anchor and positive and negative inputs, respectively, for the $i^{\mathrm{th}}$ triplet. The top layer of the branches is a 128-dimensional fully-connected layer that represents the embedding vector of its output.

In our particular case, even when FaceNet is trained with our own dataset (https://github.com/davidsandberg/facenet), we adopted the model (github.com/nyoki-mtl/keras-facenet) pretrained on the MS-Celeb-1M dataset [51], which has reported an accuracy of 99.6% for the Labeled Faces in the Wild dataset (LFW) [52]. Indeed, MS-Celeb-1M covers an extensive population of individuals, including one million faces from 100,000 users. Given that in an assistance robot scenario, we are dealing with users not included in the training, MS-Celeb-1M enriches the face space, providing a generalized dataset that fits with the requirements of a context with unpredictable facial variability for unseen identities. To adjust to the pre-trained FaceNet model, the cropped face is finally fed with a range of values between {0, 255} in the RGB color space, obtaining a feature vector *f* that corresponds to the fully-connected layer.

### Offline Performance of the Face Re-Identification Module

**Figure 4:**
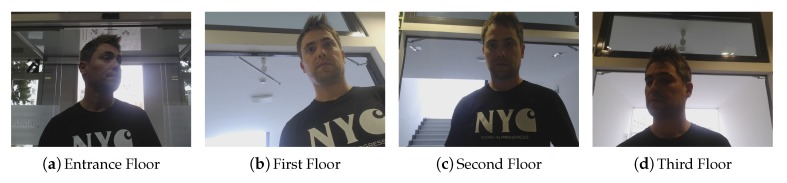
Perspectives from the different robots.

To evaluate face recognition performance, a set of images was captured using 18 volunteers at the UPV/EHU Faculty of Informatics. Four robots were used, each located on a different floor. Due to different robot morphologies, even though all robots used the same camera model (Logitech C920 HD PRO (https://www.logitech.com/en-gb/product/hd-pro-webcam-c920?crid=34) webcam) with 800 × 600 resolution, camera perspectives differed, and thus, images varied considerably. Figure 4 allows visually appreciating and comparing the different perspectives of the robots.

**Figure 5:**
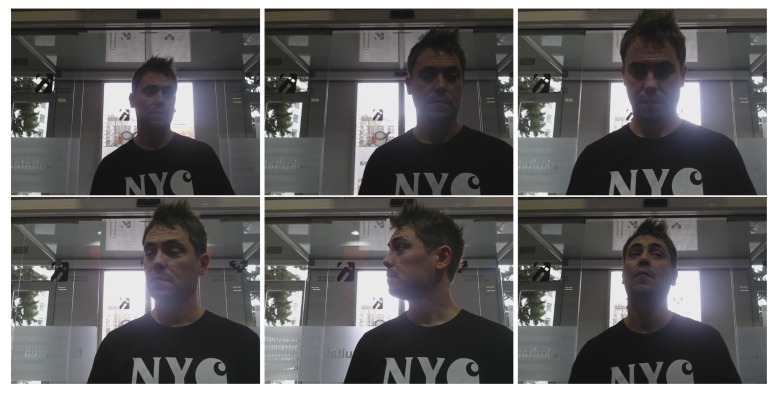
Some instances of the capturing process. The spot corresponds to the main entry of the Faculty, where the lighting conditions are critical.

Each of these individuals was placed in front of every robot and at different locations. The individual was allowed to move freely, but smoothly, with the only requirement that the head remained visible in the screen. Figure 5 shows some images corresponding to a single user in a specific spot.

**Figure 6:**
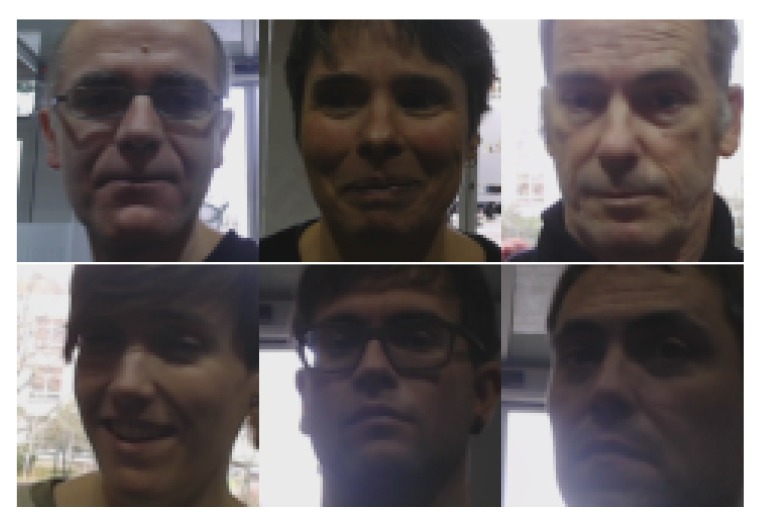
Examples of normalized detected faces, for different identities, after weak alignment and cropping of a sample per identity captured on Floor 0. Challenging pose and illuminations variations are present.

**Figure 7:**
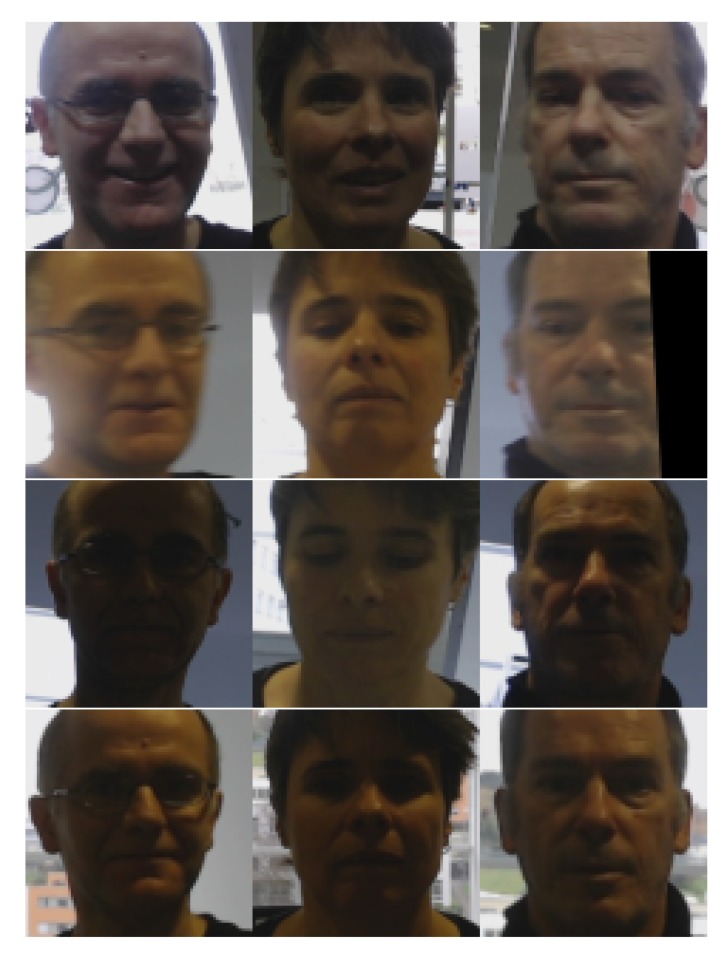
Samples after face normalization captured on different floors evidencing variations in pose and illumination across floors. Each column corresponds to a specific participant (Identities 1, 2, 3, 6, and 7) and shows one acquisition per floor.

Although images were captured at 0.5 Hz, no systematic capture procedure was followed to assure an identical number of samples per identity. Face pose was unrestricted; therefore, the dataset presented different challenges for face detectors. The total dataset contained 1808 images of the users, distributed according to Table 1. On each image, the face detector setup described above was applied, obtaining a positive detection with facial landmarks in 1316 images. No face detection failures were removed. The number of detected faces in relation to identities and floors is also indicated in brackets in Table 1. A normalized face of six identities captured on Floor 0 is shown in Figure 6, while the captures of five identities on the four floors are presented in Figure 7. The samples posed a number of challenging situations given the variations in terms of image quality, pose, and illumination.

To evaluate the approach, we adopted the focus of a re-identification problem, because each query user (probe) had to be sought in a set of user candidates (gallery). We assumed for simplicity a closed world scenario where the probe was always present in the gallery. According to the re-identification literature, the Cumulative Match Characteristics (CMC) was employed in the tables, as it is the metric most widely used for re-identification results. CMC relates the rank with the identification rate. Thus, increasing the rank would never reduce the identification rate.

In a first global experiment, the whole dataset was considered. Each occurrence of the user was taken as the probe, and the rest of the dataset composed the gallery. The results for this experiment are shown in Table 2. The accuracy obtained for rank-1 and rank-10 was respectively 97.42% and 99.01%. Given that the gallery was comprised of 18 different identities, the rank-1 random guess would report an accuracy of 5.6%. Therefore, the reported accuracy was promising. Though this experiment gave an idea about the face recognition module’s performance, it was highly optimistic because the gallery contained images of the probe (query user) taken by the same robot and on the same floor, which is not the typical situation expected in the considered scenario.

A most frequent and real situation is when the probe (query user) must be looked up in the images taken by other floors’ robots. To evaluate the proposed module in this latter scenario, again, every image captured by each robot was used as the probe, and the gallery was comprised of samples captured by a single robot, but on another floor. Considering the four floors, there were 16 combinations of probe-gallery experiments. Table 3 summarizes their respective results. As expected, the highest performance was obtained when the probe and gallery corresponded to the same floor (robot), reporting rather optimistic accuracies, similar to those presented in Table 2. The system achieved 98.5% as the maximum rank-1 score matching users from the basement floor. Instead, slightly lower results were obtained on the second floor (94.8% of rank-1). Compared to the basement captures, back light seemed to be the main factor obscuring facial details.

**Figure 8:**
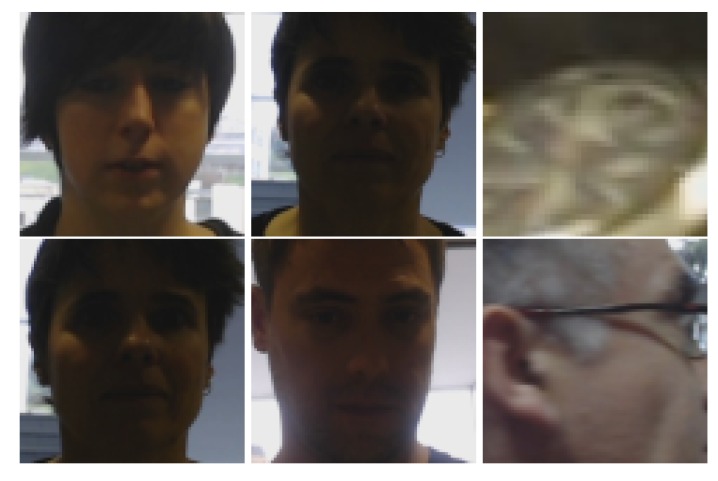
Examples of re-identification failures across floors. The upper row corresponds to probes and the bottom row to the matched image from the gallery. Observe that a face detection error is matched with another detection error.

When observing the results for the challenging scenario, where the probe and gallery were captured by different robots, i.e., different floors, 92.2% was obtained as the highest accuracy (Floor 0 for Probe 0; Floor 1 for Gallery 1); and the lowest score was 66.5%, involving the basement and third floor. Observing Table 1, there were zero detections or three identities (Floors 2 and 3). Certainly, no positive match was possible in the experiments when there was no sample for one user in the gallery. Some matching errors are presented in Figure 8. For rank-1, the total number of correct matches was 1167, while for failures 152, i.e., an accuracy of 88.48%.

**Figure 9:**
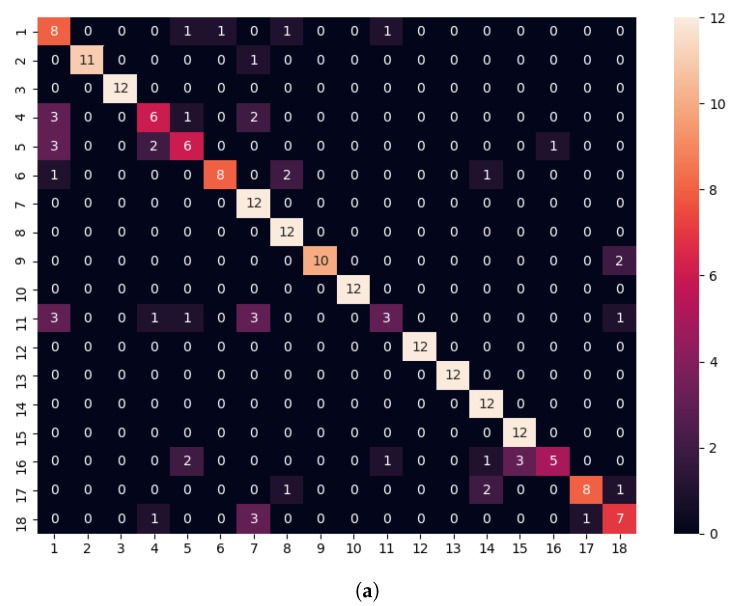

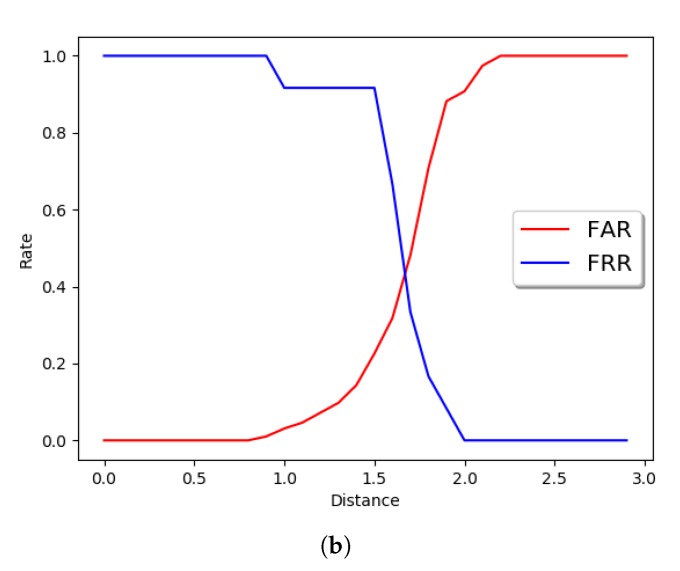
(**a**) Confusion matrix obtained for different floors’ matching. (**b**) FAR and FRR obtained.

A final experiment took advantage of the temporal information during the human–robot interaction. Indeed, the robot captured multiple instances of the individual face, and therefore, instead of matching a single shot (probe), we adopted the strategy of matching the whole set of samples for an identity on our floor, with the collection of samples captured by the robot located on the target floor. To simulate this scenario, we measured the distance on the target floor with the 18 identities in the dataset, choosing the one with the lowest distance average as the potential match. Therefore, for the set of $n_{i}$ samples of an identity *a* on the origin floor *o*, $x_{a}^{o}=\left\{s_{a,1}^{o},s_{a,2}^{o},\ldots ,s_{a,n_{i}^{o}}^{o}\right\}$ was matched against the samples of any individual met on the target floor *t*, $x_{b}^{t}=\left\{s_{b,1}^{t},s_{b,2}^{t},\ldots ,s_{b,n_{b}^{t}}^{t}\right\}$, where the target was computed by obtaining the average of the distances obtained for every sample combination making use of the sample embeddings $s\left(x_{a}^{o},x_{b}^{t}\right)=\frac{\sum_{i=1}^{n_{a}^{o}}\sum_{j=1}^{n_{b}^{t}}d\left(e\left(s_{a,i}^{o}\right),e\left(s_{b,j}^{t}\right)\right)}{n_{a}^{o}\times n_{b}^{t}}$. For this approach, Table 4 summarizes the results only for experiments where the origin and target floors were not the same. The reader may observe that rank-1 achieved an accuracy over 81%. The confusion matrix obtained for the 18 identities, considering rank-1, is shown in the left side of Figure 9, evidencing that the behavior was different across the identities. On one side, Identities 11 and 16 were poorly identified, while 4 and 5 were just correctly recognized at 50%. On the other side, eight identities were correctly matched. It could be argued that there was a shorter number of samples for some of those identities on some floors (e.g., 4, 5, and 11), a circumstance that may also be affected by the image quality. In any case, it was important to make use of an identity verification threshold for online use.

On the right side of Figure 9, the reader may observe the False Acceptance Rate (FAR) and False Rejection Rate (FRR) obtained, modifying the distance threshold to consider a positive match. Future research should consider the introduction of image quality measures and collecting larger datasets in real conditions.

## 5. Integration of the Face Recognition Module in the Multi-Robot System

As a final step, the face recognition module was adapted and integrated into GidaBot as previously shown in Figure 1. The module was linked to the multirobot_navigation node, and it was only activated when a floor change was required, i.e., when two (or more) robots were involved in the guiding task. Internally, the leading robot must take a photo of the user and send it to the robot located on the goal floor together with the information related to the start and end points of the navigation task. The second robot then was responsible for receiving the goal message and the visitor’s image and waited for the visitor that started the task. The following requirements were identified and fulfilled as described.

### 5.1. Changes on the GUI

**Figure 10:**
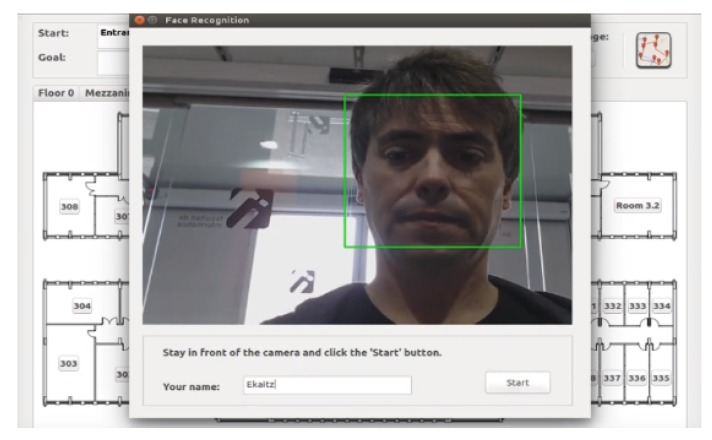
Window displayed to captured user’s image when the goal requires a floor change.

Information pop-up messages needed to be added to help the user with the procedure. Before leaving a user to be picked up by the next guide, a window showing the image to be captured was displayed, together with some helpful information describing the procedure to take a photo (see Figure 10). This pop-up message invited the user to type her/his name to be named using voice commands. In the second robot, a message was displayed first with the name of the user the robot was waiting for, and then, a window similar to the previous one was displayed. The result of the re-identification was also shown to the user.

### 5.2. Robots Must Wait for Users

The presence or absence of people in front of the camera should be distinguished. A computationally light face detector (Viola–Jones (VJ)) was used to ensure that at least one face was present on the image to be captured to avoid failures. When more than one face was available, the biggest one would be selected, as it was considered to be the closest one.

### 5.3. Face Detection Method Used

The face detection module was responsible for aligning and cropping the images before extracting FaceNet embeddings. Offline experiments were done using DLIB and MTCNN in a cascade manner. Due to the lower computational load of VJ, we decided to test the three of them to look for the more appropriate one for the problem at hand.

### 5.4. Criteria for Deciding if Two Images Correspond to the Same Person

**Figure 11:**
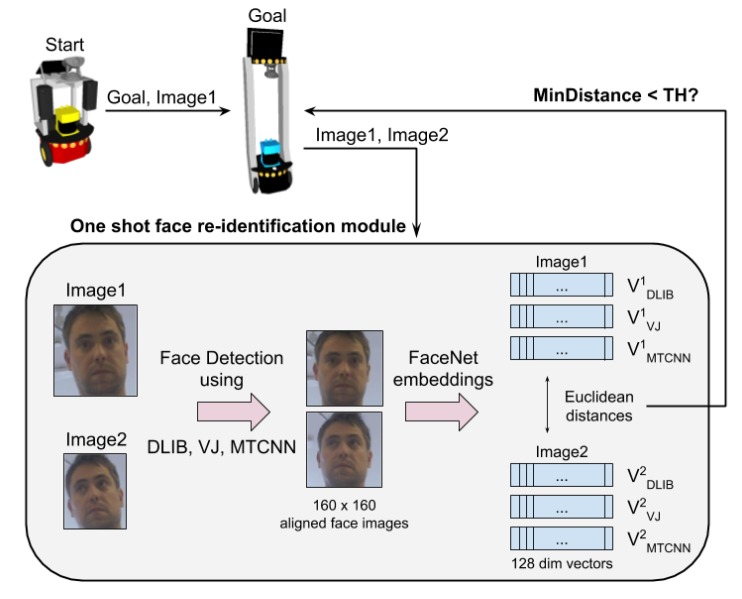
One-shot re-identification architecture in GidaBot.

Criteria must be defined to decide if two images correspond to the same person or not. Opposite the offline experimental phase, the deployed robotic system required one-shot face verification. Independent of the face detection method, as FaceNet embeddings were used as feature vectors, a threshold on the Euclidean distance between vectors should determine if those vectors from different images contained the same individual. However, preliminary experiments showed us how complex it could be to determine a threshold value that worked satisfactorily for any of the three face detection methods proposed. Thus, instead of relying just on a single method, we decided to fuse the capabilities of the different methods by computing the distance between images using the three of them (DLIB, VJ, and MTCNN) and making the final decision applying the threshold (TH) to the minimum of the three distances (MinDistance) obtained (see Figure 11).

This was the core of the practical approach presented in the paper. The results shown in Section 6 reveal the improvement introduced by this fused system.

### 5.5. Changes on the Multirobot_Navigation Node

Besides being responsible for sending the robot to the goal, each time a floor change occurred, the node must verify if the two captured images corresponded to the same identity. Note that the first image was taken by the first robot on the origin floor, and only the second capture was taken by the current robot. If after three trials, there was no positive match, the robot would in any case take the user to the destination.

### 5.6. Endorsement of the Face Identification Process

In such a distributed system, each robot should ideally be capable of giving an answer when it takes on the navigation goal. In practice, the hardware demanded deep learning processes were not satisfied by any of our mobile platforms. As a temporary solution, a notebook computer endowed with a GPU was connected to the robot network, dedicated to the face verification task.

## 6. Online Experimental Setup and Results

Offline experiments validated the face recognition module’s performance as such. However, real-world results may differ strongly from captured datasets. Therefore, the applicability and reliability of the approach in the real scenario should be tested.

To this aim, three robots were used during 10 working days for about 2 h each day, alternating mornings and afternoons, in an experiment carried out at the Faculty. People were asked to participate, requesting from one of the robots at least one guiding tour that required moving to a different floor; i.e., each guiding task involved the interaction of more than one robot.

Summarizing, fifty-six different users were involved (25 female/31 male) in the experiments.

Given the fact that according to the interaction protocol, the system guided the subject to capture his/her face when the guiding tour was requested, and later the next floor’s robot waited for the subject, who would be again guided for a new capture to verify his/her identity, therefore, impostors (or potential false positives) would rarely occur in this scenario. However, the experiment included forcing impostors by means of interrupting randomly some visitors and trying to confuse the system using volunteer impostors. After this observation, in the final experimental setup, ninety percent of the image pairs were genuine in the sense that they belonged to the same person (but images were taken by different robots). As a result, ten percent of the time, the system was confronted with images not belonging to the same person, i.e., true negatives or impostors were forced. The whole dataset was comprised of up to 199 image pairs (roughly 90% genuine and 10% impostors), which were taken during real tours and compared in real conditions.

Table 5 summarizes the results achieved for the proposed *MinDistance*. As described in Section 5, the proposal fused the embeddings obtained with up to three face detectors, to make us of the minimal distance. In the overall experiment, only nine false negatives occurred, and no false positive was reported.

**Figure 12:**
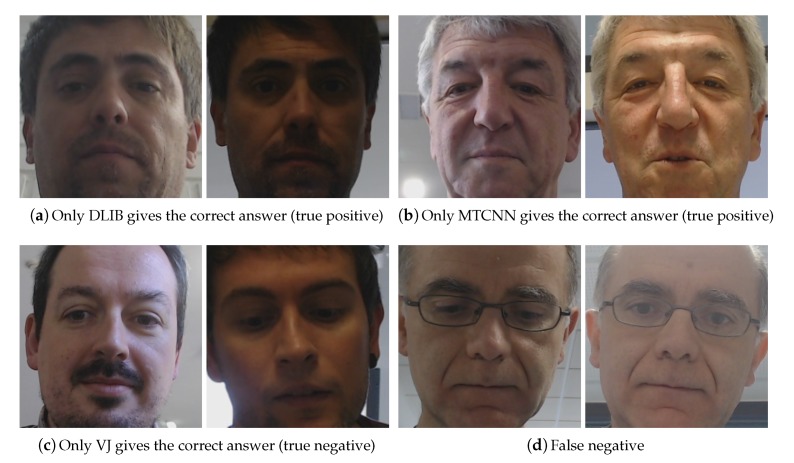
Examples of cases where at most one method gave the proper answer.

The fusion of three face detectors avoided the bias present in each one, providing a final system that was less dependent to the strengths and weaknesses of a single detector, i.e., being more adaptive to the different unrestricted conditions that were present during the real human-robot interaction. Analyzing log data to explore the importance of the different face detectors in the overall process, we observed that the DLIB face detector provided the minimal distance in most cases, about 51% of the time, while VJ and MTCNN succeeded respectively about 28% and 21% of the time. Those rates are summarized in Table 6, including the accuracy achieved using a single detector. A fast observation reveals that the best accuracy was achieved using the DLIB face detector, reaching up to 84%. In any case, it was evident that all detectors were relevant to make the fusion approach more robust, as the final accuracy of the expert fusion approach reduced the error by more than 68%. Examples of singularities related to each detector occurring during the experiments are shown in Figure 12.

Some additional information related to the robots activity during the guiding tours is summarized in Table 7. The interaction episodes were balanced among the three robots, as among the total number of 199 guiding requests, they respectively interacted 62, 76, and 61 times. Their covered distances were roughly between two and three kilometers, evidencing the real conditions of the experiment carried out.

**Figure 13:**
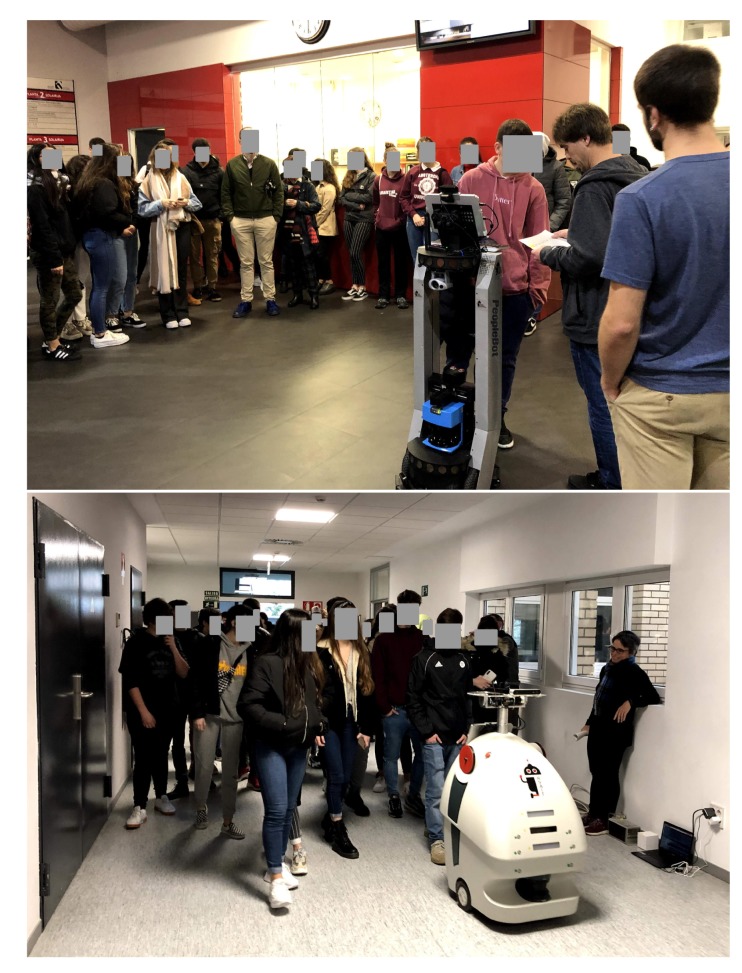
Xare 2019 event at the Faculty of Informatics.

Last but not least, it is worth highlighting that the system was operative in the real world. GidaBot has been extensively used for different exhibitions, and Figure 13 illustrates the guiding activities using two robots with the secondary school students. A video available on RSAIT’s Youtube channel https://www.youtube.com/watch?v=5c4RDQg5Rsc) shows two of our robots guiding a user from the entrance hall to a room located on the third floor of the faculty and how the user was verified by the second robot.

## 7. Conclusions and Further Work

This paper focused on the deployment and integration of a face re-identification module in a heterogeneous multi-robot social service system designed for guiding tasks over multi-floor environments, called GidaBot. The formulated problem was complex in nature. Multiple sensors and unrestricted capture and illumination conditions were present. The scenario assumed the presence of a single robot per floor, and inter-robot communication was required to provide personalized guiding tours.

A subject recognition strategy was adopted, choosing the face as the main cue. The development process required two steps. First, standard current face detectors and facial embeddings adequateness were evaluated with 1808 test images corresponding to 18 different identities (not registered) captured along four different floors. Experiments showed promising results reporting a rank-1 CMC over 81% for re-identification across different floors.

Secondly, to deploy the face recognition in the real multi-robot tour guide system, opportunistically, a slightly different strategy was taken. Instead of using a single face detector, three face detectors were simultaneously used (VJ, DLIB, and MTCNN), adopting a minimal distance approach in the face embedding representation space. During a 10 day real-life experiment, the final accuracy reached a value over 95% after assisting 56 users in 199 interaction episodes.

Compared to the offline experiments, the online performance was considerably increased. This might be partly due to the fact that the action of capturing the image was guided by the GUI. A comparison with other state-of-the-art face-based recognition techniques remains for further work. However, what really improved the efficiency of the face recognition module in the real multi-robot application was the fused approach. This improved the reported performance by 10% over the best individual accuracy given by MTCNN. The method employed showed a desirable generalization capability. Due to the nature of the task, the system was presented with faces not previously seen. The robustness over new situations was a critical condition that the developed system overcame. Which method performed better under which lighting conditions requires further experiments to be properly answered.

Even if we considered the results more than satisfactory given the complexity of the scenario, there were some factors that could currently affect the system’s performance. For instance, it suffered from WiFi connection interruptions, and there were zones that were more prone to signal looses. Those gaps affected the inter-robot communication, and an alternative to the ROS multimaster system must be found in the near future.

The assisted capture increased the sample quality, but made the system more difficult to use. Ideally, a high quality capture should be made automatically by the robot without direct human intervention, selecting the best sample(s) to model the person being guided.

Additionally, the computational requirements forced us to add an extra element that acted somehow as a centralized spot and incremented the network traffic. The current robot processing limitations could be overcome by adding a cloud-based face recognition service instead of using an extra computational unit. Alternatively, providing a Coral-like board (https://coral.ai/docs/dev-board/datasheet/) to each of the robots would regain the distributed nature of the system, as well as reduce the network traffic, making the system more robust against WiFi dropping.

Future steps should also tackle the interaction with users in a human-like manner, reducing GUI interaction. Thus, a first challenge would be to add the possibility to understand goals by voice commands. It might be worth also investigating the possibility of using a multimodal approach and combining the visual information with audio sources for person identification.

## Figures and Tables

**Table 1:** Dataset distribution of samples per identity and floor (in brackets, the final number of detected faces per identity and floor).

	Floor			
Id	0	1	2	3
1	87 (77)	29 (28)	22 (9)	45 (34)
2	41 (38)	16 (12)	19 (10)	35 (35)
3	27 (26)	22 (17)	26 (20)	21 (14)
4	35 (31)	21 (11)	20 (0)	27 (3)
5	19 (18)	18 (12)	24 (2)	37 (0)
6	22 (22)	6 (4)	21 (15)	41 (13)
7	25 (23)	14 (12)	21 (18)	16 (15)
8	27 (27)	20 (12)	22 (21)	36 (28)
9	24 (23)	22 (17)	20 (12)	26 (16)
10	16 (16)	24 (21)	21 (20)	26 (26)
11	32 (19)	44 (24)	22 (2)	31 (0)
12	22 (22)	23 (21)	22 (10)	21 (18)
13	26 (20)	12 (12)	21 (20)	28 (21)
14	25(25)	25 (19)	20 (15)	31 (25)
15	17 (17)	24 (22)	26 (22)	26 (23)
16	27 (27)	24 (24)	21 (15)	25 (12)
17	25 (24)	24 (19)	27 (14)	28 (19)
18	20(19)	10 (8)	13 (6)	25 (17)

**Table 2:** Rank scores from 1 to 10 for users on all the building’s floors.

Rank-1	Rank-2	Rank-3	Rank-4	Rank-5	Rank-6	Rank-7	Rank-8	Rank-9	Rank-10
97.42	97.95	98.33	98.56	98.64	98.71	98.71	98.79	98.94	99.01

**Table 3:** Rank-1, Rank-5, and Rank-10 scores for each floor compared to other floors.

	Gallery				
	Floor	0	1	2	3
Probe	0	98.5/98.9/98.9	92.2/92.2/92.2	77.4/77.4/77.4	80.7/81.7/81.9
	1	91.5/91.5/92.2	98.3/98.3/98.6	83.5/84.1/85.2	80.7/81.1/81.1
	2	69.3/69.3/69.7	83.5/84.4/84.4	94.8/96.5/97.8	75.8/77.1/80.2
	3	66.5/67.4/68.3	72.7/72.7/73.0	76.3/76.6/80.1	97.8/99.1/99.4

**Table 4:** Rank scores from 1 to 10 considering multiple samples per identity and different floors.

Rank-1	Rank-2	Rank-3	Rank-4	Rank-5	Rank-6	Rank-7	Rank-8	Rank-9	Rank-10
81.16	86.96	88.89	91.79	93.72	93.72	96.14	96.62	96.62	97.10

**Table 5:** Performance obtained in the multi-robot system.

	Accuracy	Sensitivity	Specificity	F1
MinDistance	0.95	0.95	1	0.97

**Table 6:** Performance values of each method individually.

	Accuracy	MinDistance %
DLIB	0.84	51.26
VJ	0.693	28.0
MTCNN	0.70	20.74

**Table 7:** Robots’ overall trip info.

	Total Distance (m)	Floor Changes
ROBOT 1	2313	62
ROBOT 2	2757	76
ROBOT 3	1973	61
